# The Substitution of Soybean Lecithin With Lysophospholipids Promoted the Growth Performance, Lipid Metabolism, and Reduced Dietary Lipid Inclusion for Pacific White Shrimp (*Litopenaeus vannamei*)

**DOI:** 10.1155/anu/6301061

**Published:** 2026-01-18

**Authors:** Yang Xiao, Xiaoqin Li, Shenghao Li, Weida Wu, Lili Lei, Hongfei Huang, Xiangjun Leng

**Affiliations:** ^1^ National Demonstration Center for Experimental Fisheries Science Education, Shanghai Ocean University, Shanghai, 201306, China, shou.edu.cn; ^2^ Research Centre of the Ministry of Agriculture and Rural Affairs on Environmental Ecology and Fish Nutrition, Shanghai Ocean University, Shanghai, 201306, China, shou.edu.cn; ^3^ Louis Dreyfus(Shanghai)Animal Nutrition Technology Co. Ltd., Shanghai, 201306, China

**Keywords:** growth performance, lipid metabolism, lysophospholipid, Pacific white shrimp, soybean lecithin

## Abstract

This study evaluated the feasibility of replacing soybean lecithin (SBL) with lysophospholipids (LYLs) in the diet of Pacific white shrimp, *Litopenaeus vannamei*. Five isonitrogenous diets were formulated as the control diet with 15 g/kg soybean oil and 15 g/kg SBL inclusion (LYL‐0), three SBL‐substituted diets with 1/3, 2/3, and 3/3 of SBL substituted with the same amounts of LYL (LYL‐0.5, LYL‐1, and LYL‐1.5), and a low‐lipid diet (LYL‐L) with 5 g/kg soybean oil and 15 g/kg LYL inclusion. Then, shrimp (1.6 ± 0.1 g) were fed with the above diets for 8 weeks. The LYL‐1.5 group presented significantly higher weight gain (WG) than the control (*p* < 0.05), but no significant difference was observed in WG and feed conversion ratio (FCR) between the control and LYL‐L group, or between the LYL‐L and LYL‐1.5 group (*p* > 0.05). Whole‐body lipid levels were found to be substantially increased in the LYL‐1 and LYL‐1.5 groups compared to the control, while hepatopancreatic lipid content and lipid droplet area (Oil Red O stain) were significantly lower in the LYL‐1, LYL‐1.5, and LYL‐L groups (*p* < 0.05). Serum high‐density lipoprotein cholesterol (HDL‐C), lipoprotein lipase (LPL), hepatic lipase activities, and the apparent digestibility of crude lipid were significantly increased in the LYL‐1 and LYL‐1.5 groups (*p* < 0.05). In contrast, serum triglyceride (TG) and total cholesterol (TC) levels were significantly decreased in the LYL‐1.5 and LYL‐L groups (*p* < 0.05). In summary, under the present conditions, the complete replacement of SBL with LYLs improved growth performance and lipid metabolism and enabled a 10 g/kg reduction in dietary lipid level for Pacific white shrimp.

## 1. Introduction

Lipids in the diet serve as a crucial supply of essential fatty acids for aquatic species, which play key roles in maintaining growth performance and metabolic stability of animals [1]. Higher lipid content in feeds will increase the cost of aquaculture, but lower lipid content may also negatively affect shrimp growth [2].

Phospholipids are important lipid molecules maintaining cellular integrity and functionality, facilitating lipid emulsification, and enhancing the intestinal absorption of long‐chain fatty acids [3]. Moreover, as an essential dietary component for crustaceans, phospholipids contribute significantly to the regulation of growth and overall health. Notably, the physiological responses elicited by phospholipids are dose‐dependent and vary according to the specific molecular forms [4]. Soybean lecithin (SBL) is commonly used as the primary source of phospholipids and emulsifiers in commercial shrimp diets with inclusion levels ranging from 1% to 3% [5]. SBL has been shown to enhance the growth performance, stress resistance, and survival rates in fish and crustaceans [6]. However, certain studies have indicated that adding SBL to the diet had no notable impact on nutrient digestibility or growth outcomes [7–10]. These findings suggest that the inclusion of SBL may not elicit the expected positive effects on growth performance under certain conditions.

Lysophospholipids (LYL) are the enzymatic hydrolysates of phospholipids by phospholipase A1 or A2, resulting in a molecular structure distinct from that of SBL in phosphatidyl substituent groups and fatty acid composition [11, 12]. Characterized by a single fatty acid tail, LYLs exhibit higher hydrophilicity. The unique property can disperse lipids more efficiently and enhance lipid emulsification to form smaller micelles, thereby facilitating lipid absorption and transport [13]. Research has demonstrated that LYLs possess approximately five times greater emulsifying capacity for dietary lipids compared to other phospholipids [14].

The inclusion of 0.1% LYLs in the diet of Atlantic salmon (*Salmo salar*) increased the final body weight by 5% and significantly enhanced the structural integrity and metabolic activity of intestinal cells with promoting hepatic metabolism of lipids, carbohydrates, and amino acids [13]. Likewise, in largemouth bass (*Micropterus salmoides*), substituting 50% of soybean oil with enzymatically hydrolyzed SBL led to improved growth performance and nutrient utilization, while also enhancing antioxidant capacity and promoting stability in the intestinal microbiota [15]. The phospholipid requirement of *Litopenaeus vannamei* varies with growth stage, environmental conditions, and diet composition. For the juvenile and subadult Pacific white shrimps, the appropriate dietary level was reported to be 10.3 g/kg and 6.5 g/kg, respectively [16]. However, the application of LYLs in crustacean diets is limited, which has been reported in giant tiger shrimp (*Penaeus monodon*), oriental river prawn (*Macrobrachium nipponense*) and Pacific white shrimp (*Litopenaeus vannamei*) [5, 17, 18]. These investigations focused on growth performance and lipid metabolism, while less on nutrient digestibility and hepatopancreas fatty acid composition.

While LYL has shown the potential in enhancing growth and lipid metabolism, it remains unclear whether they can completely replace SBL in the diet of *L. vannamei*. Given their superior emulsifying properties, we hypothesize that LYL can effectively substitute SBL and allow for a reduction in dietary lipid level without negatively affecting growth, nutrient digestibility, or lipid metabolism. Therefore, the present study investigated the effects of replacing various levels of SBL with LYL and the potential of decreasing soybean oil inclusion in the diet of *L. vannamei*, when LYL completely replaced SBL, including the growth performance, lipid metabolism, apparent nutrient digestibility, and hepatopancreatic fatty acid composition. The findings will direct the application of LYL in shrimp diets.

## 2. Materials and Methods

### 2.1. Experimental Diet

A control diet (LYL‐0) was formulated to contain 15 g/kg soybean oil and 15 g/kg SBL, then LYLs were used to replace 1/3, 2/3, and all (3/3) of dietary SBL at inclusion levels of 5, 10, and 15 g/kg (LYL‐0.5, LYL‐1, and LYL‐1.5 diets), respectively, resulting in SBL levels of 10, 5, and 0. Based on the LYL‐1.5 diet, the inclusion level of soybean oil was reduced from 15 to 5 g/kg to form the low lipid diet (LYL‐L). Thus, five isonitrogenous diets were formulated in the present study. Y_2_O_3_ was included as a marker to detect nutrient digestibility. Solid components were milled and filtered using an 80‐mesh sieve before being uniformly blended. After the water was added, the mixtures were processed into sinking pellets (2 mm) using a single‐screw extruder. The pellets were post‐cooked at 95°C for 25 min, then dried and stored in sealed containers until use. The LYLs used in this study were provided by Louis Dreyfus (Shanghai) Animal Nutrition Technology Co., Ltd., which contained 48 g/kg lysophosphatidylcholine (LPC). The formulation, proximate composition, and fatty acid profile of the experimental diets are shown in Tables 1 and 2.

**Table 1:** Formulation and proximate composition of experimental diets (air‐dry basis, g/kg).

Ingredients	LYL‐0	LYL‐0.5	LYL‐1	LYL‐1.5	LYL‐L
Fish meal	160.0	160.0	160.0	160.0	160.0
Poultry meal	80.0	80.0	80.0	80.0	80.0
Cottonseed protein concentrate	40.0	40.0	40.0	40.0	40.0
Wheat flour	254.9	254.9	254.9	254.9	264.9
Soybean meal	210.0	210.0	210.0	210.0	210.0
Soy protein concentrate	60.0	60.0	60.0	60.0	60.0
Corn gluten meal	40.0	40.0	40.0	40.0	40.0
Peanut meal	60.0	60.0	60.0	60.0	60.0
Squid visceral meal	40.0	40.0	40.0	40.0	40.0
Ca(H_2_PO_4_)_2_	20.0	20.0	20.0	20.0	20.0
Soybean oil	15.0	15.0	15.0	15.0	5.0
Soyabean lecithin	15.0	10.0	5.0	0.0	0.0
Lysophospholipids	0.0	5.0	10.0	15.0	15.0
Vitamin^a^ and mineral premix^b^	1.2	1.2	1.2	1.2	1.2
Choline chloride	2.0	2.0	2.0	2.0	2.0
KCl	1.0	1.0	1.0	1.0	1.0
Ascorbyl phosphate	0.4	0.4	0.4	0.4	0.4
Y_2_O_3_	0.5	0.5	0.5	0.5	0.5
Total	1000.0	1000.0	1000.0	1000.0	1000.0
Proximate composition^c^	—	—	—	—	—
Moisture	86.1	63.2	73.8	71.0	70.1
Crude protein	422.4	427.3	420.7	423.8	427.3
Crude lipid	69.7	69.0	70.1	68.6	58.9
Crude ash	80.5	82.3	81.9	80.9	86.1

^a^Vitamin premix (g or IU/kg), vitamin A, 250 000 IU; vitamin D3, 190 000 IU; vitamin E, 1 500 IU; vitamin K3, 0.45 g; vitamin B1, 1 g; vitamin B2, 1.5 g; vitamin B6, 1 g; nicotinic acid, 2 g; D, calcium pantothenate; 2 g; and folic acid, 0.1 g.

^b^Mineral premix (mg or g/kg), Cu, 2 g; Fe, 37 g; Mn, 3.9 g; Zn, 5.9 g; Se, 19 mg; I, 0.78 g; Co, 78 mg.

^c^The proximate composition of the experimental diets were measured.

**Table 2:** Fatty acid composition of experimental diets (percentage of total fatty acids, %).

Parameters	LYL‐0	LYL‐0.5	LYL‐1	LYL‐1.5	LYL‐L
C14:0	3.29	3.30	3.18	2.61	2.89
C15:0	0.30	0.29	0.27	0.28	0.29
C16:0	24.02	23.83	24.42	24.87	24.05
C17:0	0.61	0.63	0.62	0.56	0.45
C18:0	6.33	6.07	6.55	6.77	7.27
C20:0	0.60	0.59	0.62	0.58	0.63
SFAs	35.15	34.71	35.65	35.66	35.62
C16:1	2.44	2.49	2.36	2.54	3.12
C17:1	0.56	0.56	0.59	0.62	0.62
C18:1*n* – 7	2.31	2.31	2.42	2.25	2.23
C18:1*n* – 9	23.19	23.13	22.28	21.91	22.64
C20:1	0.85	0.78	0.80	0.74	0.79
C22:1	0.28	0.23	0.28	0.26	0.23
MUFAs	29.64	29.51	28.74	28.32	29.63
C18:2*n* – 6	21.92	22.14	21.91	22.40	21.96
C20:2*n* – 6	0.71	0.69	0.68	0.69	0.61
C20:3*n* – 6	0.13	0.11	0.13	0.10	0.11
C18:3*n* – 6	0.19	0.18	0.16	0.12	0.17
C20:4*n* – 6 ARA	0.87	0.86	0.92	0.84	0.95
*n* – 6 PUFAs	23.81	23.98	23.80	24.15	23.80
C18:3*n* – 3 ALA	2.81	2.84	3.08	3.26	2.79
C20:5*n* – 3 EPA	4.08	4.01	4.35	4.28	3.85
C22:6*n* – 3 DHA	3.57	3.89	4.12	4.09	3.52
*n* – 3 PUFAs	10.47	10.73	11.55	11.63	10.15
PUFAs	34.28	34.71	35.35	35.78	33.95
*n* – 3/*n* – 6 PUFAs	0.44	0.45	0.49	0.48	0.43

### 2.2. Shrimp and Experimental Design

The juvenile shrimp were purchased from Shanghai Zhangxian Aquatic Professional Cooperative (Shanghai, China) and transported to the indoor cement tanks at the Binhai aquaculture farm (Shanghai, China) for acclimation. The shrimp were fed a commercial diet (crude protein ≥440 g/kg and crude lipid ≥50 g/kg) for 40 days. Then, 1000 juvenile shrimp (initial body weight 1.6 ± 0.1 g) were selected and randomly assigned to five experimental groups with four replicates and 50 shrimp per replicate.

Throughout the 56‐day feeding trial, shrimp were fed four times daily at 07:00, 12:00, 17:00, and 23:00. Feed allocation was determined based on body weight and subsequently adjusted according to water temperature and observed feeding activity, which was 8% in the early stage, 5% in the mid‐stage, and 3% in the late stage of the trial. All the diets were ensured to be consumed within 2 h after feeding. Continuous aeration was provided in all cages, and about one‐third of the water was renewed weekly. Waste and feces were removed using the siphon method. Water quality was monitored daily to maintain the following conditions: temperature 26–32°C, salinity 0.5‰–1.0‰, dissolved oxygen ≥5.0 mg/L, ammonia nitrogen <0.1 mg/L, and nitrite <0.1 mg/L.

### 2.3. Sample Collection

Before the feeding trial commenced, 100 shrimp were randomly selected for the assessment of initial whole‐body proximate composition. During the final 2 weeks of the trial, fecal samples were collected from the experimental cages within 2 h post‐feeding using a siphoning technique. Intact fecal strands were meticulously isolated, promptly frozen at −20°C, and later analyzed to determine the apparent digestibility coefficients (ADCs) of crude protein and lipid.

At the conclusion of the 56‐day feeding trial, shrimp were subjected to a 24‐h fasting period. All shrimp in each cage were counted and bulk weighed to calculate survival, weight gain (WG), and feed conversion ratio (FCR). Three shrimp per replicate were collected and stored at −20°C for final whole‐body proximate composition analysis. Based on this data, protein retention efficiency (PRE) and lipid retention efficiency (LRE) were calculated. Additionally, four shrimp per replicate were measured body length, body weight, and hepatopancreas weight to calculate hepatosomatic index (HSI) and condition factor (CF). Five shrimp per replicate were drew hemolymph from the pericardial cavity, then stored at 4°C for 12 h, and centrifuged at 4000 *r*/min for 10 min. The supernatant was collected and stored at −80°C for analysis of serum biochemical parameters.

Furthermore, six shrimp per replicate were used to sample hepatopancreas and then stored at −80°C for analysis of proximate composition, fatty acid profile and digestive enzyme activity. An additional four shrimp per replicate were sampled hepatopancreas, which was fixed in Bouin’s solution for histological examination.

### 2.4. Measurement Indicators and Methods

#### 2.4.1. Growth Performance

WG (%) = (Final mean body weight − initial mean body weight)/initial mean body weight × 100

Survival (%) = (Final number of shrimp/initial number of shrimp) × 100

FCR = Dry weight of feed intake (FI)/(final body weight − initial body weight)

PRE (%) = Protein gain/protein intake × 100

LRE (%) = Lipid gain/lipid intake × 100

FI (g/shrimp) = Total FI/final number of shrimp

Hepatopancreas somatic index (HSI, %) = Hepatopancreas weight/body weight × 100

CF (g/cm^3^) = Body weight/(body length)^3^ × 100

Meat yield (%) = Muscle weight/total body weight × 100

#### 2.4.2. Proximate Composition of Diets, Whole Body, Hepatopancreas, and Feces

The proximate composition of the samples was analyzed following the standard methods of the Association of Official Analytical Chemists (AOAC) [19]. Moisture content was assessed by drying samples at 105°C until a constant weight was achieved. Crude protein levels were analyzed using a Kjeldahl autoanalyzer (Model 2300, Foss Tecator, Sweden). Lipid content was extracted and quantified following the chloroform–methanol method. Ash content was determined by combusting the samples in a muffle furnace at 550°C for 6 h.

#### 2.4.3. Serum Biochemical Parameters

Serum biochemical parameters included triglyceride (TG), total cholesterol (TC), high‐density lipoprotein cholesterol (HDL‐C), low‐density lipoprotein cholesterol (LDL‐C), lipoprotein lipase (LPL), total antioxidant capacity (T‐AOC), malondialdehyde (MDA), albumin (ALB), and glucose (GLU). All parameters were determined using commercial assay kits strictly following the manufacturer’s instructions (Nanjing Jiancheng Bioengineering Institute, Nanjing, China).

#### 2.4.4. Digestive Enzymes and Fatty Acid Composition of the Hepatopancreas

The hepatopancreas tissues were defrosted at 4°C and subsequently blended with physiological saline at a ratio of 1:9 (w/v). The resulting mixtures were subjected to centrifugation at 2500 rpm for 10 min. The obtained supernatant fractions were then utilized for measuring the activities of various digestive enzymes, namely protease, amylase, and lipase. All enzyme assays were conducted using commercial kits following the manufacturer’s instructions (Nanjing Jiancheng Bioengineering Institute, Nanjing, China).

Fatty acid composition of the hepatopancreas was analyzed following the method described by Yang et al. [20].

#### 2.4.5. Histological Analysis of the Hepatopancreas and Intestine

The hepatopancreatic tissue was embedded in OCT compound, rapidly frozen, and sectioned into 8–10 μm thick frozen slices, followed by treatment with 60% isopropanol. The tissue slices underwent staining using a 0.5% Oil Red O solution for 10 min. Following background elimination, hematoxylin was employed to counterstain the nuclei, and glycerin jelly was used to mount the slides for observing lipid deposits. Morphological features of the hepatopancreas were examined and photographed using a Nikon‐YS100 microscope (Nikon, Japan). Quantitative analysis of Oil Red O‐stained sections was performed using Image‐Pro Plus 6.3 software under the same threshold settings (0–130). The percentage of stained area was used as an index of lipid accumulation in the hepatopancreas.

#### 2.4.6. Nutrient Utilization Efficiency

Yttrium (Y) levels in dietary and fecal samples were quantified by high‐performance liquid chromatography combined with inductively coupled plasma mass spectrometry (HPLC‐ICP‐MS; Vista MPX, Varian, Palo Alto, California, USA).

The ADCs of crude protein and lipid were calculated using the following formula:

Apparent digestibility of protein (or lipid, %) = 100 × [1 − (*Y* in diet × nutrient in feces)/(*Y* in feces × nutrient in diet)]

The retention efficiency of nutrients was calculated as follows:

PRE (%) = Protein gain/protein intake × 100

LRE (%) = Lipid gain/lipid intake × 100

#### 2.4.7. Statistical Analysis

Data are expressed as mean ± standard deviation. Statistical differences among groups were assessed using one‐way analysis of variance (One‐way ANOVA) to determine whether overall significant differences existed. Tukey’s post hoc multiple comparison test was employed for pairwise comparisons between groups. A significance level of *p*  < 0.05 was considered statistically significant. All statistical analyses were performed using SPSS version 26.0 (SPSS Inc., Chicago, IL, USA).

## 3. Results

### 3.1. Growth Performance

As shown in Table 3, with the increasing level of LYL replacing SBL, an increasing trend was observed in WG. Specifically, the WG in the LYL‐1.5 group was significantly increased by 8.24% compared to the control (*p* < 0.05), while the FCR decreased by 0.07 (*p* > 0.05). The replacement of SBL with LYLs had no significant effects on survival, FCR, HSI, CF, and meat yield (*p* > 0.05). Additionally, no significant differences were observed in any growth performance parameters between the LYL‐L group and the control, or between the LYL‐L group and the LYL‐1.5 groups (*p* > 0.05).

**Table 3:** Effects of lysophospholipids replacing soybean lecithin on growth performance of *Litopenaeus vannamei*.

Parameters	LYL‐0	LYL‐0.5	LYL‐1	LYL‐1.5	LYL‐L
IBW (g)	1.60 ± 0.10	1.60 ± 0.10	1.60 ± 0.10	1.60 ± 0.10	1.60 ± 0.10
FBW (g)	17.66 ± 0.18^a^	18.18 ± 0.08^ab^	18.32 ± 0.44^ab^	18.98 ± 0.64^b^	18.39 ± 0.70^ab^
WG (%)	1003.6 ± 11.2^a^	1036.0 ± 5.4^ab^	1045.0 ± 27.7^ab^	1086.3 ± 40.3^b^	1049.2 ± 44.1^ab^
Survival (%)	91.00 ± 4.16	88.00 ± 1.63	90.50 ± 1.91	92.50 ± 5.00	92.50 ± 3.00
FCR	1.30 ± 0.03	1.31 ± 0.04	1.30 ± 0.04	1.23 ± 0.06	1.25 ± 0.04
HSI (%)	4.01 ± 0.37	3.93 ± 0.22	3.88 ± 0.12	3.52 ± 0.35	3.82 ± 0.19
CF (g cm^3^)	0.83 ± 0.05	0.81 ± 0.06	0.82 ± 0.05	0.82 ± 0.08	0.81 ± 0.06
Meat yield (%)	52.98 ± 1.55	51.53 ± 1.22	51.80 ± 0.22	53.09 ± 1.70	51.90 ± 0.55

*Note:* Values in the same column with different lowercase superscript letters indicate significant differences (*p* < 0.05), as below.

Abbreviations: CF, condition factor; FBW, final body weight; FCR, feed conversion ratio; HIS, hepatosomatic index; IBW, initial body weight; WG, weight gain.

### 3.2. Proximate Composition of Whole Body and Hepatopancreas

Compared with the control group, the crude lipid content of whole shrimp was significantly increased in the LYL‐0.5, LYL‐1, and LYL‐1.5 groups (*p* < 0.05), while the crude lipid content of the hepatopancreas was significantly decreased in the LYL‐1 and LYL‐1.5 groups (*p* < 0.05). The crude lipid content of whole shrimp in the LYL‐L group was similar to the control group (*p* > 0.05) and was significantly lower than that in the LYL‐1.5 group (*p* < 0.05). Additionally, the LYL‐L group presented significantly lower hepatopancreatic crude lipid content than the control group (*p* < 0.05). There were no significant differences in the moisture, crude protein, and ash contents of whole shrimp and hepatopancreas among all the treatments (*p* > 0.05; Table 4).

**Table 4:** Effects of lysophospholipids replacing soybean lecithin on the proximate composition of whole body and hepatopancreas of *Litopenaeus vannamei* (fresh weight, g/kg).

Parameters	LYL‐0	LYL‐0.5	LYL‐1	LYL‐1.5	LYL‐L
Whole body					
Moisture	744.52 ± 9.31	743.25 ± 13.05	746.39 ± 3.41	745.83 ± 8.84	745.58 ± 8.89
Crude protein	186.56 ± 8.42	193.74 ± 9.63	190.60 ± 4.50	194.78 ± 3.69	187.58 ± 3.90
Crude lipid	13.93 ± 1.40^a^	16.43 ± 0.85^bc^	16.88 ± 1.53^bc^	17.65 ± 0.86^c^	15.00 ± 0.80^ab^
Crude ash	25.65 ± 1.91	26.90 ± 0.42	25.70 ± 0.38	25.35 ± 0.50	25.65 ± 0.21
Hepatopancreas					
Moisture	644.34 ± 17.30	651.87 ± 5.97	641.81 ± 14.70	659.98 ± 20.87	651.75 ± 23.38
Crude protein	131.13 ± 6.88	132.63 ± 3.44	138.23 ± 3.75	134.50 ± 2.83	138.67 ± 4.67
Crude lipid	50.27 ± 3.97^b^	51.42 ± 4.69^b^	38.08 ± 2.89^a^	38.19 ± 4.64^a^	43.38 ± 1.74^a^
Crude ash	12.65 ± 0.98	13.22 ± 0.29	14.44 ± 1.26	13.08 ± 1.38	13.82 ± 0.89

*Note:* Values in the same column with different lowercase superscript letters indicate significant differences (*p* < 0.05).

### 3.3. Serum Biochemical Indicators

In Table 5, serum GLU, HDL‐C, and LPL levels were significantly higher in the LYL‐1 and LYL‐1.5 groups (*p* < 0.05), while TC, TG, and LDL‐C levels were significantly lower than those in the control group (*p* < 0.05). Additionally, the ALB level in the LYL‐1.5 group was significantly higher than that in the other four groups (*p* < 0.05). In antioxidant capacity, the LYL‐1 and LYL‐1.5 groups showed significantly lower MDA levels and significantly higher T‐AOC than the control (*p* < 0.05).

**Table 5:** Effects of lysophospholipids replacing soybean lecithin on serum biochemical indexes of *Litopenaeus vannamei*.

Parameters	LYL‐0	LYL‐0.5	LYL‐1	LYL‐1.5	LYL‐L
ALB (g/L)	3.60 ± 0.33^a^	3.63 ± 0.35^a^	4.09 ± 0.38^ab^	6.29 ± 0.44^c^	4.48 ± 0.23^b^
GLU (mmol/L)	0.54 ± 0.07^a^	0.55 ± 0.06^ab^	0.62 ± 0.04^b^	0.63 ± 0.05^b^	0.55 ± 0.05^ab^
TG (mmol/L)	1.42 ± 0.16^b^	1.30 ± 0.09^ab^	1.23 ± 0.07^a^	1.17 ± 0.09^a^	1.25 ± 0.10^a^
TC (mmol/L)	0.33 ± 0.04^b^	0.35 ± 0.03^b^	0.26 ± 0.02^a^	0.23 ± 0.03^a^	0.26 ± 0.02^a^
HDL‐C (mmol/L)	0.32 ± 0.03^a^	0.36 ± 0.03^ab^	0.37 ± 0.02^bc^	0.41 ± 0.03^c^	0.41 ± 0.03^c^
LDL‐C (mmol/L)	0.25 ± 0.02^b^	0.25 ± 0.03^b^	0.22 ± 0.02^a^	0.21 ± 0.01^a^	0.23 ± 0.01^ab^
MDA (nmol/mL)	0.74 ± 0.05^b^	0.72 ± 0.06^b^	0.63 ± 0.06^a^	0.64 ± 0.05^a^	0.69 ± 0.05^ab^
LPL (μmol/ml)	6.08 ± 0.52^a^	9.23 ± 0.54^c^	10.10 ± 0.88^c^	9.75 ± 0.65^c^	7.39 ± 0.32^b^
T‐AOC (nmol/mL)	130.84 ± 8.62^a^	138.81 ± 10.28^ab^	150.96 ± 8.24^b^	148.98 ± 6.83^b^	134.79 ± 10.17^a^

*Note:* Values in the same column with different lowercase superscript letters indicate significant differences (*p* < 0.05).

Abbreviations: ALB, albumin; GLU, glucose; HDL‐C, high‐density lipoprotein cholesterol; LDL‐C, low‐density lipoprotein cholesterol; LPL, lipoprotein lipase; MDA, malondialdehyde; T‐AOC, total antioxidant capacity; TC, total cholesterol; TG, triglycerides.

When dietary lipid level was reduced by 10 g/kg, the LYL‐L group exhibited significantly higher serum ALB, HDL‐C, and LPL levels, and significantly lower TG and TC levels than the control group (*p* < 0.05). However, ALB and T‐AOC levels in the LYL‐L group were significantly lower than those in the LYL‐1.5 group (*p* < 0.05).

### 3.4. Hepatopancreatic Digestive Enzyme Activities and Glycogen Content

As shown in Table 6, the activities of hepatopancreatic protease (except the LYL‐L group) and lipase were significantly elevated in all LYL groups compared to the control group (*p* < 0.05). Additionally, amylase activity in the LYL‐1 and LYL‐1.5 groups was significantly higher than that in the control group (*p* < 0.05). In glycogen content, the LYL‐1.5 group exhibited significantly higher levels than the other four groups (*p* < 0.05). In protease, amylase activity and glycogen content, the LYL‐L group showed similar levels to the control groups (*p* > 0.05) and significantly lower levels than the LYL‐1.5 group (*p* < 0.05). No significant difference was observed in lipase activity between the LYL‐L and LYL‐1.5 groups (*p* > 0.05).

**Table 6:** Effects of lysophospholipids replacing soybean lecithin on digestive enzymes and glycogen contents in hepatopancreas of *Litopenaeus vannamei*.

Parameters	LYL‐0	LYL‐0.5	LYL‐1	LYL‐1.5	LYL‐L
Protease (U/g)	453.84 ± 41.51^a^	523.31 ± 47.07^b^	560.65 ± 41.33^bc^	594.23 ± 34.18^c^	500.24 ± 45.82^ab^
Amylase (U/g)	101.40 ± 2.99^a^	109.23 ± 7.55^a^	131.35 ± 7.02^b^	128.25 ± 5.62^b^	104.21 ± 5.02^a^
Lipase (U/g)	312.84 ± 14.56^a^	366.27 ± 18.57^b^	377.65 ± 16.14^b^	371.36 ± 20.22^b^	365.31 ± 17.03^b^
Glycogen (mg/g)	6.29 ± 0.55^a^	6.59 ± 0.50^a^	6.48 ± 0.46^a^	8.36 ± 0.61^b^	5.77 ± 0.38^a^

*Note:* The results in the table are calculated according to the fresh weight of samples. Values in the same column with different lowercase superscript letters indicate significant differences (*p* < 0.05).

### 3.5. Fatty Acid Composition of the Hepatopancreas

Compared with the control group, the relative proportions of C16:0, total saturated fatty acids (SFAs), C18:1*n*–7, C20:3*n* – 6 and C18:3*n* – 6 in the hepatopancreas of LYL‐1.5 group were significantly decreased (*p* < 0.05), while the proportion of α‐linolenic acid (ALA) was significantly increased (*p* < 0.05). In the LYL‐L group, the proportions of C16:0, SFAs, and C17:1 were significantly lower than those in the control group (*p* < 0.05), while the proportions of C15:0, C17:0, C20:1, C20:3*n* – 6, and C18:3*n* – 6 were significantly higher than those in the LYL‐1.5 group (*p* < 0.05). No significant differences were observed in the MUFAs, *n* – 3 PUFAs, *n* – 6 PUFAs or PUFAs among the five groups (*p* > 0.05; Table 7).

**Table 7:** Effects of lysophospholipids replacing soybean lecithin on fatty acid composition in hepatopancreas of *Litopenaeus vannamei* (percentage of total fatty acids, %).

Parameters	LYL‐0	LYL‐0.5	LYL‐1	LYL‐1.5	LYL‐L
C14:0	0.52 ± 0.02	0.55 ± 0.02	0.50 ± 0.03	0.46 ± 0.02	0.50 ± 0.07
C15:0	0.39 ± 0.04^ab^	0.33 ± 0.02^a^	0.35 ± 0.03^ab^	0.34 ± 0.02^a^	0.42 ± 0.04^b^
C16:0	19.84 ± 0.99^b^	19.73 ± 0.42^b^	18.67 ± 0.80^ab^	17.09 ± 0.60^a^	17.86 ± 0.39^a^
C17:0	0.69 ± 0.03^ab^	0.66 ± 0.04^a^	0.59 ± 0.04^a^	0.62 ± 0.04^a^	0.77 ± 0.05^b^
C18:0	9.97 ± 0.38	9.49 ± 0.62	8.61 ± 0.59	8.55 ± 0.79	8.58 ± 0.71
C20:0	0.58 ± 0.04	0.56 ± 0.04	0.52 ± 0.04	0.51 ± 0.04	0.53 ± 0.06
C22:0	0.52 ± 0.06	0.50 ± 0.03	0.53 ± 0.04	0.52 ± 0.04	0.48 ± 0.04
SFAs	32.51 ± 0.82^c^	31.81 ± 0.53^bc^	29.78 ± 1.26^ab^	28.08 ± 0.98^a^	29.14 ± 0.96^a^
C14:1	0.41 ± 0.02	0.45 ± 0.05	0.42 ± 0.02	0.41 ± 0.03	0.37 ± 0.04
C16:1	2.61 ± 0.34	2.97 ± 0.29	2.95 ± 0.15	2.59 ± 0.26	2.65 ± 0.14
C17:1	0.90 ± 0.03^b^	0.81 ± 0.02^a^	0.76 ± 0.03^a^	0.78 ± 0.02^a^	0.81 ± 0.02^a^
C18:1*n* – 7	3.35 ± 0.12^b^	3.26 ± 0.31^ab^	2.75 ± 0.23^ab^	2.67 ± 0.13^a^	3.14 ± 0.28^ab^
C18:1*n* – 9	19.45 ± 1.18	19.07 ± 0.97	19.59 ± 0.99	20.35 ± 1.18	19.17 ± 1.02
C20:1	1.43 ± 0.08^ab^	1.39 ± 0.02^ab^	1.34 ± 0.03^ab^	1.32 ± 0.03^a^	1.50 ± 0.11^b^
MUFAs	28.16 ± 0.98	27.94 ± 0.78	27.81 ± 0.87	28.12 ± 1.11	27.64 ± 0.66
C18:2*n* – 6	17.28 ± 0.79	18.03 ± 1.14	17.81 ± 0.71	18.29 ± 0.87	16.75 ± 0.79
C20:2*n* – 6	1.29 ± 0.07	1.26 ± 0.06	1.32 ± 0.06	1.36 ± 0.05	1.38 ± 0.12
C20:3*n* – 6	0.55 ± 0.02^b^	0.51 ± 0.05^ab^	0.49 ± 0.03^ab^	0.45 ± 0.05^a^	0.57 ± 0.04^b^
C18:3*n* – 6	0.13 ± 0.01^b^	0.14 ± 0.01^b^	0.11 ± 0.01^ab^	0.09 ± 0.02^a^	0.14 ± 0.02^b^
C20:4*n* – 6 ARA	2.79 ± 0.10	2.79 ± 0.13	2.88 ± 0.22	2.55 ± 0.23	2.95 ± 0.14
*n* – 6 PUFAs	22.05 ± 0.75	22.73 ± 1.16	22.60 ± 0.46	22.74 ± 0.92	21.79 ± 0.87
C18:3*n* – 3 ALA	0.56 ± 0.02^a^	0.61 ± 0.02^ab^	0.61 ± 0.02^ab^	0.65 ± 0.03^b^	0.59 ± 0.04^ab^
C20:5*n* – 3 EPA	8.45 ± 0.33	8.26 ± 0.37	8.93 ± 0.74	8.35 ± 0.41	8.49 ± 0.39
C22:6*n* – 3 DHA	8.23 ± 0.55	8.56 ± 0.38	8.43 ± 0.62	8.50 ± 0.51	8.19 ± 0.83
*n* – 3 PUFAs	17.24 ± 0.23	17.43 ± 0.37	17.97 ± 0.57	17.50 ± 0.87	17.27 ± 1.25
PUFAs	39.29 ± 0.70	40.16 ± 1.07	40.57 ± 0.51	40.25 ± 0.34	39.05 ± 2.12
*n* – 3/*n* – 6 PUFAs	0.78 ± 0.04	0.77 ± 0.05	0.79 ± 0.04	0.77 ± 0.06	0.79 ± 0.03

### 3.6. Nutrient Utilization

In Table 8, the ADC of crude lipid and LRE in the LYL‐1 and LYL‐1.5 groups, together with the PRE in the LYL‐1.5 groups, were significantly increased compared to the control group (*p* < 0.05). However, replacing SBL with LYLs showed no significant effect on the ADC of crude protein (*p* > 0.05). The LYL‐L group presented, significantly higher ADC of crude lipid than the control group (*p* < 0.05) and significantly lower ADC of crude protein than the LYL‐1.5 group (*p* < 0.05).

**Table 8:** Effects of lysophospholipids replacing soybean lecithin on nutrient utilization of *Litopenaeus vannamei* (%).

Parameters	LYL‐0	LYL‐0.5	LYL‐1	LYL‐1.5	LYL‐L
ADCP	84.45 ± 1.94^ab^	85.05 ± 1.67^ab^	86.89 ± 0.61^ab^	87.81 ± 1.73^b^	84.13 ± 1.47^a^
ADCL	84.22 ± 1.85^a^	86.66 ± 1.38^ab^	90.70 ± 1.24^c^	90.44 ± 1.91^c^	90.27 ± 1.90^bc^
PRR	30.45 ± 1.42^a^	32.29 ± 0.30^ab^	32.38 ± 0.93^ab^	34.67 ± 1.25^b^	32.75 ± 0.42^ab^
LRR	13.06 ± 0.50^a^	17.26 ± 0.88^b^	17.03 ± 1.51^b^	17.24 ± 0.90^b^	15.18 ± 1.36^ab^

*Note:* Values in the same column with different lowercase superscript letters indicate significant differences (*p* < 0.05).

Abbreviations: ADCL, apparent digestibility coefficient of crude lipid; ADCP, apparent digestibility coefficient of crude protein; LRR, lipid retention rate; PRR, protein retention rate.

### 3.7. Histology of the Hepatopancreas

Figure 1 shows the Oil Red O‐stained sections of hepatopancreas. The red‐stained areas represent lipid droplets. With the increasing replacement of SBL by LYLs, the lipid droplet area in hepatopancreas tended to decrease, and the LYL‐1, LYL‐1.5, and LYL‐L groups showed significantly lower lipid droplet areas than the control group (*p* < 0.05). No significant difference was observed between the LYL‐L and LYL‐1.5 groups (*p* > 0.05).

Figure 1Hepatopancreas Oil Red O staining section of *Litopenaeus vannamei* (200×). *Note*: (A): LYL‐0 group; (B): LYL‐0.5 group; (C): LYL‐1 group; (D): LYL‐1.5 group; (E): LYL‐L group; (F): dyeing area of lipid droplets, bar charts bearing different lowercase superscript letters indicate statistically significant differences (*p* < 0.05).

**Figure:**
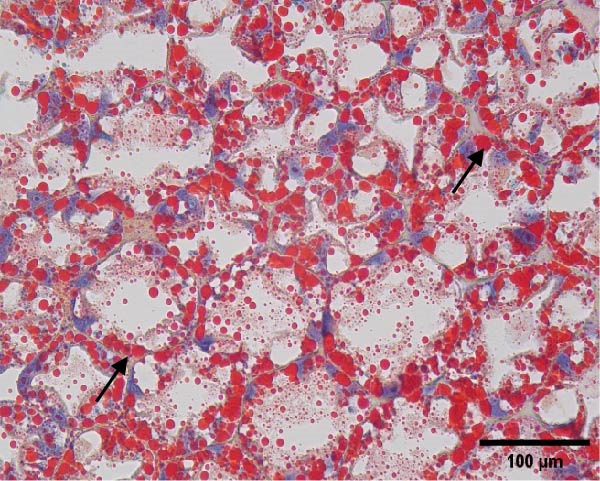
(A)

**Figure:**
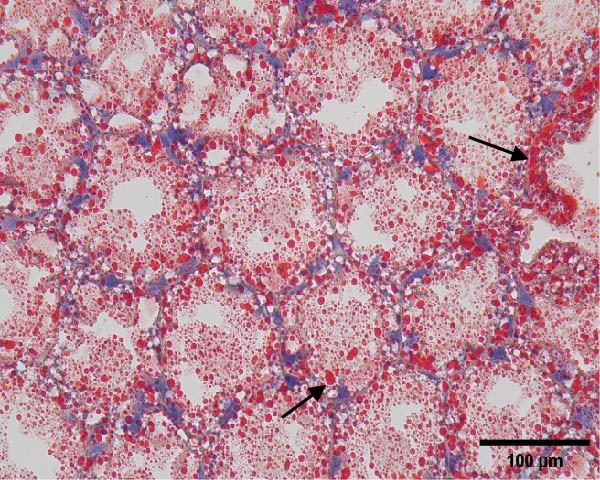
(B)

**Figure:**
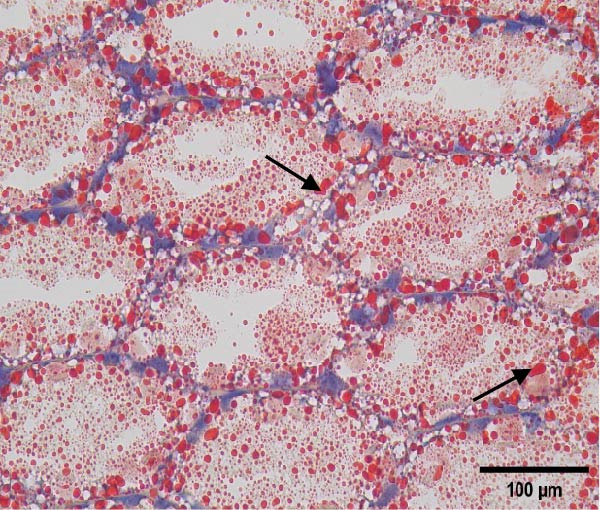
(C)

**Figure:**
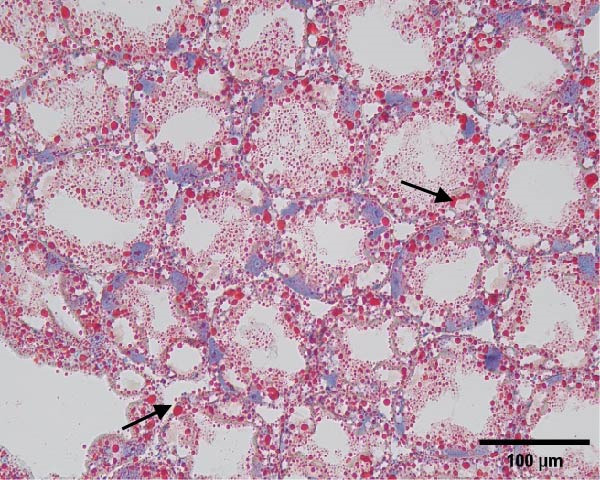
(D)

**Figure:**
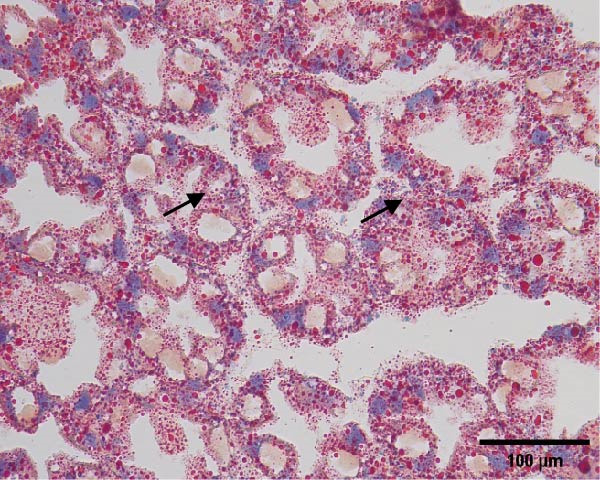
(E)

**Figure:**
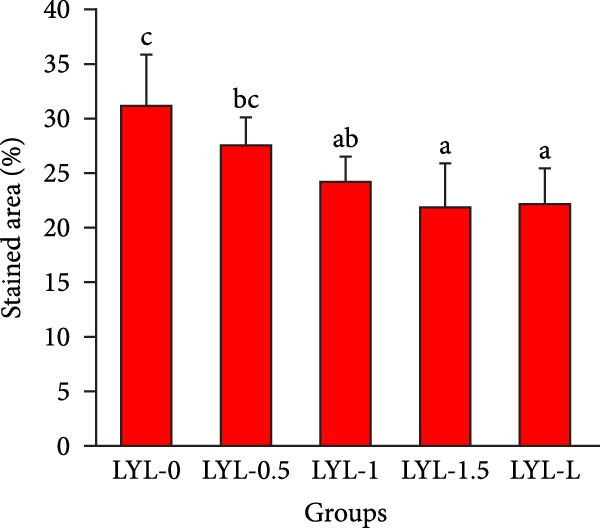
(F)

## 4. Discusssion

### 4.1. Effects of Replacing SBL With LYLs on the Growth Performance of *Litopenaeus vannamei*

In a study on Nile tilapia (*Oreochromis niloticus × O. mossambicus*), dietary supplementation with LYLs enhanced the transcellular transport rate of macromolecules across the intestinal epithelium, thereby promoting lipid digestion and absorption, which significantly improved WG and reduced the FCR, without effect on the survival [21]. Similarly, Weng et al. [22] demonstrated that dietary inclusion of LYLs significantly enhanced WG and decreased FCR in large yellow croaker (*Larimichthys crocea*). In the present study, the complete replacement of SBL with LYLs significantly improved the WG of *L. vannamei* and numerically decreased FCR. Although this study did not examine the expression of lipid transporter and lipase‐related genes, the previous studies in largemouth bass have suggested that LYLs enhanced lipid utilization by upregulating genes such as LPL and CPT1 [23].

In the present study, LYL supplementation also enhanced lipid metabolic capacity, as indicated by increasing the key lipid metabolism enzyme activities and reducing the lipid accumulation in hepatopancreas. These mechanisms, together with elevated digestive enzyme activity and improved apparent lipid digestibility, may have collectively contributed to the improved growth performance and feed efficiency of *L. vannamei*.

The growth‐promoting effect of LYLs appears to be dose‐dependent. Previous research has shown that dietary supplementation of 0.2% lysophospholipid significantly suppressed the growth of largemouth bass [23]. This may be because high inclusion of LYLs exhibits cytotoxic effects by disrupting the integrity of phospholipid bilayers in the cell membrane, and induces oxidative stress via the overproduction of endogenous reactive oxygen species. Contrary to these findings, the current study demonstrated that LYL inclusion level of 15 g/kg still promoted the growth of *L. vannamei*, which may be attributed to species‐specific differences in lipid metabolism and tolerance.

In crustaceans, the hepatopancreas serves as the main site for lipid storage and is crucial for both lipid metabolism and immune function. However, excessive lipid deposition in this organ can disrupt lipid homeostasis and oxidative balance, potentially compromising immune responses [24]. According to Bao et al. [25] adding 300 mg/kg of LYLs to the diet effectively reduced liver lipid levels in largemouth bass. Similarly, the inclusion of 2 g/kg LYLs in the diet led to an increase in whole‐body crude lipid content in rainbow trout [26].

In the present study, when 2/3 or 3/3 of SBL was replaced by LYLs, the crude lipid content in the hepatopancreas of *L. vannamei* was significantly decreased, while the whole‐body crude lipid content was significantly increased. This may be attributed to the enhanced lipid metabolism and transport capacity in the hepatopancreas induced by LYLs, which facilitated the mobilization of excess lipids from the hepatopancreas to the muscle for storage [27, 28]. In Figure 1, the lipid droplet area (oil red‐stained area) significantly decreased in the two groups, confirming that LYL supplementation effectively inhibited lipid deposition in the hepatopancreas and promoted lipid metabolism.

### 4.2. Effects of Replacing SBL With LYLs on Nutrient Utilization in *Litopenaeus vannamei*

This study demonstrated that replacing SBL with LYLs notably elevated digestive enzyme activities in the hepatopancreas of *L. vannamei*. Specifically, the activities of protease, amylase, and lipase were significantly increased in the LYL‐1 and LYL‐1.5 groups compared to the control. The promoting effect may be attributed to the amphiphilic properties of LYLs, which can improve cell membrane permeability and promote chylomicron formation, thereby increasing the contact area between chyme and digestive enzymes and ultimately enhancing enzymatic activity [29]. In Nile tilapia, El‐Sayed et al. [30] found that dietary phospholipids could enhance lipid emulsification and digestion, improve fatty acid utilization, facilitate lipid absorption and transport, and promote lipoprotein synthesis. Weng et al. [22] reported that dietary supplementation with 0.4% LYLs significantly increased the intestinal activities of protease, amylase and lipase in large yellow croaker. In addition, the inclusion of 0.1% LYLs in the diet of largemouth bass also significantly elevated hepatic lipase, amylase and total protease activities [31]. These studies supported the positive role of LYLs in enhancing digestive enzyme activity in aquatic animals.

In animals, the digestion and absorption of dietary lipids require emulsification and incorporation into micelles in the gastrointestinal tract [32]. Due to the absence of one fatty acid chain, LYLs possess strong hydrophilicity, allowing them to disperse more easily in aqueous environments to form small oil droplets, and enhance emulsion stability, thereby improving the efficiency of lipid digestion and absorption [33]. Zhang et al. [34] reported that dietary LYLs improved the ADC of crude lipid in yellowtail kingfish (*Seriola lalandi*). Similarly, Khan et al. [17] reported that giant tiger prawn (*Penaeus monodon*) fed with LYLs‐supplemented diet exhibited significantly higher WG and lipid digestibility than the fish fed with a SBL‐supplemented diet regardless of the lipid source. In this study, the LYL‐1.5 group showed significantly higher ADC of crude lipid and lipid retention compared to the control group. However, the studies about the effects of LYLs on nutrient digestibility in *L. vannamei* remain limited, and the specific underlying mechanisms warrant further investigation.

### 4.3. Effects of Replacing SBL With LYLs on Antioxidant Capacity and Lipid Metabolism in *Litopenaeus vannamei*

MDA, a final product of lipid peroxidation, is widely recognized as a biomarker for oxidative stress–induced damage in organisms. High MDA level indicates great oxidative stress caused by free radicals. In contrast, T‐AOC reflects the overall antioxidant status of organism, and it is widely used as an indicator of systemic antioxidant defense [35]. In red swamp crayfish (*Procambarus clarkii*), the inclusion of LYLs in high‐lipid diets significantly reduced hemolymph MDA content, while increased the reduced glutathione level [36]. In the present study, the serum T‐AOC levels were significantly increased, and MDA levels were significantly decreased when 2/3 or 3/3 of the SBL was replaced by LYLs, suggesting that the antioxidant capacity was enhanced in shrimp. The antioxidant‐promoting effect of LYLs may be related to the choline group in the molecular structure, which has been shown to regulate cellular redox balance and suppress inflammation, thereby improving the organism’s antioxidant capacity [37, 38].

Serum TG level is considered as an important indicator reflecting metabolic status in aquatic animals, and the elevated TG usually reflects metabolic disturbances or potential health problem [39]. In this study, substituting SBL with LYLs markedly influenced serum lipid parameters and the activities of enzymes associated with lipid metabolism. In particular, the LYL‐1.5 group showed significantly reduced levels of TG, TC, and LDL‐C, alongside significantly increased HDL‐C levels. As effective emulsifiers, LYLs may facilitate the rapid clearance of chylomicrons in blood or delay their release into the blood, thereby reducing serum TG level [40]. In addition, the elevated HDL‐C level will help to remove excess cholesterol from tissues and blood, contributing to lipid homeostasis [23]. LYLs also can promote the digestion, absorption, and transport of dietary lipids, enhance energy utilization, lipid deposition, and effectively reduce LDL‐C level [41]. Wang et al. [18] found that adding LYLs to the diet significantly lowered serum TG, TC, and LDL‐C levels in *L. vannamei*. Zhu et al. [42] also observed the decreased serum TG and LDL‐C levels by LYLs supplementation (0.05%) in high‐lipid diets for black seabream (*Acanthopagrus schlegelii*). The results of the present study indicated that the complete replacement ofSBL with LYLs enhanced lipid emulsification and metabolism in *L. vannamei*, thereby contributing to the regulation of cholesterol and lipid metabolic balance.

LPL hydrolyzes TG to provide energy for peripheral tissues, thereby regulating blood lipid metabolism [43]. In the present study, the replacement of SBL with LYLs significantly increased serum LPL activity in the present study. Generally, LPL activity increases with the increasing dietary lipid levels [44]. However, the diets used in the present study were iso‐lipidic except for the LYL‐L group. Considering the significant improvement in crude lipid apparent digestibility observed in the LYL‐1 and LYL‐1.5 groups, it is speculated that the replacement of SBL with LYLs enhanced lipid digestion and absorption through the superior emulsifying properties, thereby inducing the increase of LPL activity. In addition, LPL can hydrolyze TG in chylomicrons, thereby reducing serum TG levels, which may explain the significant decrease in serum TG concentrations observed in the LYL‐1 and LYL‐1.5 groups. Similarly, Lu et al. [45] reported that the supplementation of LYL in low‐protein, low‐lipid diets significantly enhanced hepatic LPL activities in largemouth bass. The similar results have also been observed in turbot [7].

An increased proportion of SFAs in animal tissues has been associated with the enhanced hepatic lipid accumulation [46]. In the present study, the proportion of C16:0 and SFAs in the hepatopancreas of *L. vannamei* was significantly reduced in the LYL‐1.5 group. The reduction in SFAs proportion was consistent with the observed decreases in lipid‐stained area and crude lipid content in the hepatopancreas, suggesting that LYL supplementation may enhance lipid metabolism and alleviate hepatic lipid deposition. Similarly, Xu et al. [47] reported that dietary inclusion of 0.1% and 0.25% LYLs significantly reduced hepatic SFA levels in turbot. A similar result was also observed in large yellow croaker [22]. However, the studies on the effects of LYLs on tissue fatty acid profiles in aquatic animals remain limited, and the underlying mechanisms have not been fully elucidated. Further investigation is needed to clarify the metabolic pathways.

### 4.4. The Sparing‐Effect of LYLs for Dietary Lipid

LYLs are efficient emulsifiers, with approximately five times of emulsification capacity than conventional phospholipids [14]. In the present study, dietary lipid inclusion was reduced from 15 to 5 g/kg, but the reduction did not negatively affect the growth performance of *L. vannamei* in the LYL‐L group, even significantly promoted the hepatopancreatic lipase activity and the ADC of crude lipid compared to the control group. The present results may be attributed to the superior emulsifying capacity of LYLs, which likely enhanced lipid digestion and absorption efficiency, thereby maintaining sufficient energy supply for growth [15, 29]. Additionally, hepatic glycogen content in the LYL‐L group did not differ significantly from that of the control, indicating that LYL supplementation in a low‐lipid diet still ensured adequate energy availability for metabolism.

In the present study, the absence of a low‐lipid SBL control group (SBL‐L) was a limitation, but using a single control group effectively highlighted the lipid‐sparing effect of LYLs. The comparison between the LYL‐L and control groups reflected the combined effects of lipid reduction and phospholipid source. While this design introduced some confounding factors, it still provided valuable insights into the potential of LYLs to reduce dietary lipids without compromising growth. Future studies should be conducted with an SBL‐L group to better separate these effects.

Consistent with our findings, Lu et al. [45] reported that LYL inclusion in low‐lipid diets had no negative impact on the growth of largemouth bass, and even improved the CF, whole‐body protein content, and intestinal digestive enzyme activities. In Nile tilapia, supplementing 0.1% LYLs to a low lipid diet (a reduction of 10 g/kg soybean oil) significantly reduced the FCR without significant effect on WG and survival [21]. The current results demonstrated that LYLs can effectively compensate for the reduced dietary lipid level by improving lipid digestibility and energy utilization efficiency, thereby maintaining the growth performance of *L. vannamei*. The present findings highlighted the application potential of LYLs in low‐lipid aquafeeds.

## 5. Conclusion

In summary, in a shrimp diet with 15 g/kg SBL and 15 g/kg soybean oil addition, the complete replacement of SBL with LYLs improved the WG, apparent digestibility of crude lipid, protein retention, and lipid retention of *L. vannamei*. Meanwhile, the complete replacement enhanced lipid metabolic capacity and antioxidant status while reducing lipid deposition in the hepatopancreas. Furthermore, the complete replacement of SBL with LYLs could reduce dietary soybean oil level by 10 g/kg without compromising performance.

## Conflicts of Interest

The authors declare no conflicts of interest.

## Author Contributions

**Xiangjun Leng and Yang Xiao:** methodology, conceptualization, investigation, software, validation, data curation, writing – original draft preparation, writing – review and editing. **Shenghao Li**: visualization. **Weida Wu, Lili Lei, and Hongfei Huang**: resources, supervision, formal analysis. **Xiaoqin Li**: project administration, funding acquisition.

## Funding

This research was funded by the National Key R&D Program of China (Grant 2024YFD2402005).

## Data Availability

The data that support the findings of this study are available from the corresponding author upon reasonable request.
